# Regulation and modulation of biogenic amine neurotransmission in *Drosophila* and *Caenorhabditis elegans*

**DOI:** 10.3389/fphys.2023.970405

**Published:** 2023-02-16

**Authors:** Katarzyna D. Rosikon, Megan C. Bone, Hakeem O. Lawal

**Affiliations:** Neuroscience Program, Department of Biological Sciences, Delaware State University, Dover, DE, United States

**Keywords:** synaptic release, dopamine, octopamine, 5HT, neurotransmission, *Drosophila*, *C. elegans*

## Abstract

Neurotransmitters are crucial for the relay of signals between neurons and their target. Monoamine neurotransmitters dopamine (DA), serotonin (5-HT), and histamine are found in both invertebrates and mammals and are known to control key physiological aspects in health and disease. Others, such as octopamine (OA) and tyramine (TA), are abundant in invertebrates. TA is expressed in both *Caenorhabditis elegans* and *Drosophila melanogaster* and plays important roles in the regulation of essential life functions in each organism. OA and TA are thought to act as the mammalian homologs of epinephrine and norepinephrine respectively, and when triggered, they act in response to the various stressors in the fight-or-flight response. 5-HT regulates a wide range of behaviors in *C. elegans* including egg-laying, male mating, locomotion, and pharyngeal pumping. 5-HT acts predominantly through its receptors, of which various classes have been described in both flies and worms. The adult brain of *Drosophila* is composed of approximately 80 serotonergic neurons, which are involved in modulation of circadian rhythm, feeding, aggression, and long-term memory formation. DA is a major monoamine neurotransmitter that mediates a variety of critical organismal functions and is essential for synaptic transmission in invertebrates as it is in mammals, in which it is also a precursor for the synthesis of adrenaline and noradrenaline. In *C. elegans* and *Drosophila* as in mammals, DA receptors play critical roles and are generally grouped into two classes, D1-like and D2-like based on their predicted coupling to downstream G proteins. *Drosophila* uses histamine as a neurotransmitter in photoreceptors as well as a small number of neurons in the CNS. *C. elegans* does not use histamine as a neurotransmitter. Here, we review the comprehensive set of known amine neurotransmitters found in invertebrates, and discuss their biological and modulatory functions using the vast literature on both *Drosophila* and *C. elegans*. We also suggest the potential interactions between aminergic neurotransmitters systems in the modulation of neurophysiological activity and behavior.

## 1 Introduction

Neurotransmitters play an essential role in a myriad of biological functions across different life forms from invertebrates to mammals. They serve primarily as vehicles for neuronal communication in both the Peripheral Nervous System (PNS) and the Central Nervous System (CNS). This communication relies in part on vesicular fusion mediated by Ca^2+^ influx, causing neurotransmitters to spill out into the synapse (Rizo, 2018). Expression of transmitter and receptor subtypes is critical for the development of synapses and neuronal networks, regulating development *in utero* and molecular mechanisms underlying behaviors throughout adulthood (Herlenius and Lagercrantz, 2004).

The prevalent monoamine neurotransmitters in invertebrates are 5-HT, DA, OA, and TA. Histamine is expressed in *Drosophila* but unlikely to be synthesized in *C. elegans* (Chase and Koelle, 2007; Haas et al., 2008). These aminergic neuroactive molecules modulate life functions, including learning and memory, circadian rhythms, aggression, mating behavior, thermoregulation, and many other homeostatic mechanisms (Blenau and Baumann, 2001). Monoaminergic biosynthesis pathways in *C. elegans* and *Drosophila* are often conserved with one another and with mammals, as this is the case with DA, TA, and OA (Hare and Loer, 2004; Neckameyer and Weinstein, 2005; Roeder, 2005). In *Drosophila*, histamine, the predominant neurotransmitter in photoreceptor cells, is synthesized *de novo* from L-histidine (Han et al., 2022) and recycled from glia into photoreceptors (True et al., 2005).

In part due to their essential functions, neurotransmitter levels are tightly regulated and at multiple levels. Pre-synaptically, biosynthesis, transport, release, and recycling are mediated by proteins, which are themselves subject to regulation. In invertebrates, that regulation is mediated by neuromodulatory systems which involve the histaminergic, serotonergic, dopaminergic, tyraminergic, and octopaminergic pathways (Avery and Krichmar, 2017).

Key to the action of certain neurotransmitters like DA are autoreceptors, which modulate synaptic transmission through feedback mechanisms that control neurotransmitter release and through clearance by monoamine transporters (Formisano et al., 2020). Here, we seek to describe the modulatory and regulatory roles of the different neurotransmitters and their effects in invertebrates with a focus on *Drosophila* and *C. elegans* and their respective contributions to our understanding of the mechanisms of action of these small molecules.

## 2 Dopamine

As in humans, DA is an essential monoamine in the nervous system of the fruit fly, where it mediates a variety of behaviors and physiological processes such as locomotor activity, sensory processing, cognition, courtship, egg-laying, and the reward pathway, evident in response to cocaine, nicotine, and alcohol (Bainton et al., 2000). After release, DA is recycled into the presynaptic terminal in *Drosophila* by DA transporter dDAT (Kume et al., 2005). Moreover, like in *Drosophila*, DA mediates important biological functions in *C. elegans*. The hermaphrodite possesses eight paired DA neurons: four cephalic neurons (CEP) neurons, paired left/right and dorsal/ventral in the nose tip. Left/right neuronal pairs innervate the cuticle of the head and tail, namely anterior deirid (ADE) and posterior deirid (PDE) neurons, respectively (Sulston et al., 1975). Male worms have six additional DA neurons, located in the tail, that are necessary for mating (Sulston et al*.*, 1975). DA is also synthesized in socket cells of the male tail (LeBoeuf et al., 2014). It plays several critical roles as a regulator and modulator of biological activities necessary for the worm’s survival, including locomotion, egg-laying, and foraging (Weinshenker et al., 1995; Chase et al., 2004; Hills et al., 2004; McDonald et al., 2007; Omura et al., 2012).

### 2.1 DA metabolism and neuronal regulation

#### 2.1.1 Synthesis and transport

**FIGURE 1:**
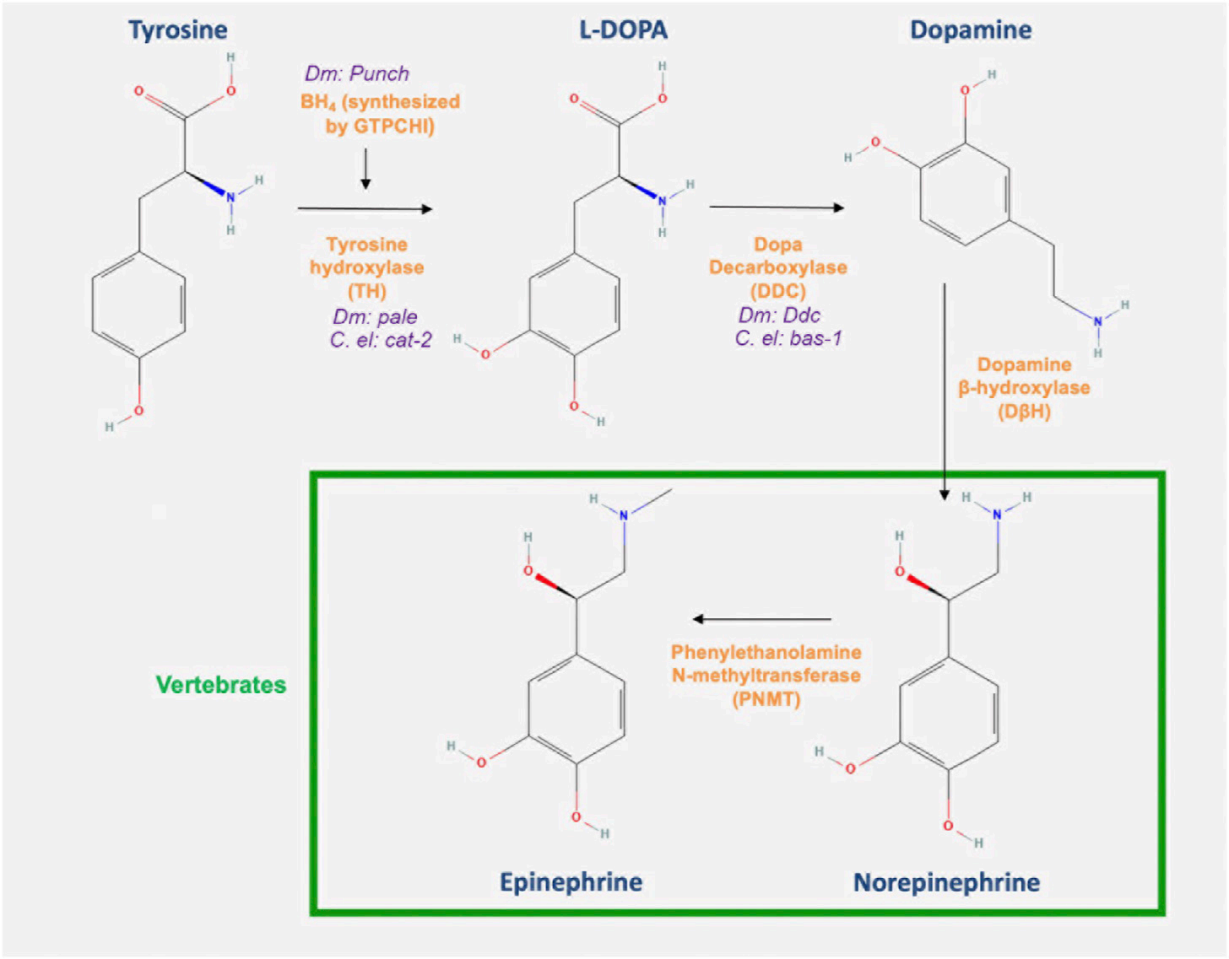
DA biosynthesis pathway. Dopamine biosynthesis is initiated by the conversion of tyrosine to L-DOPA by tyrosine hydroxylase, the first and rate limiting enzyme in the pathway. Dopa decarboxylase (DDC) then converts L-DOPA to dopamine in a terminal reaction in invertebrates. However, the pathway continues in vertebrates with the synthesis of norepinephrine and epinephrine (All chemical structures were retrieved and modified from Pubchem; https://pubchem.ncbi.nlm.nih.gov/).

There is remarkable conservation in the DA biosynthesis pathway. Like in mammals, DA is synthesized in *Drosophila* from the amino acid tyrosine in a tightly regulated pathway (Figure 1). In the first and rate limiting step, tyrosine is converted into L-3,4-dihydroxyphenylalanine (L-DOPA) by tyrosine hydroxylase (TH), which is encoded by *pale* (Neckameyer and Quinn, 1989; Neckameyer and White, 1993) (See Table 1 for a list of relevant gene names and the proteins that they code for). Crucial to this step is the regulatory cofactor tetrahydrobiopterin (BH_4_), which is synthesized by GTP cyclohydrolase I (GTPCH), the first and rate limiting enzyme in that process (Figure 1) and encoded by *Punch* in *Drosophila* (Mackay and O'Donnell, 1983; Weisberg and O'Donnell, 1986)*.* The *Punch* locus itself encodes multiple splice variants (McLean et al., 1993). The availability of BH_4_ is a key determinant in the ability of TH to synthesize DA, thus serving as a crucial checkpoint for DA synthesis writ large (Bowling et al., 2008). The second step is through the enzymatic reaction of the aromatic amino acid decarboxylase (AADC), encoded by the DOPA decarboxylase gene (*Ddc*), which converts L-DOPA into DA (Karam et al., 2020), (for review, see (Deshpande et al., 2020)). The tight regulation of DA homeostasis extends well beyond synthesis. After it is produced, DA is quickly shuttled into synaptic vesicles by the vesicular monoamine transporter (VMAT), encoded by the *VMAT* gene in *Drosophila* (Deshpande et al., 2020). VMAT is an antiporter that exchanges H^+^ for one molecule of DA in an ATP dependent process (for review, see (Deshpande et al., 2020)). Indeed, DA synthesis is coupled to its translocation into synaptic vesicles, such that there is likely a complex comprising both the DA synthesis and transport machinery working together in close proximity to maintain that tight DA regulation (Cartier et al., 2010; Wang et al., 2011). Also, crucial to this complex is the protein Catecholaminesup (encoded by *Catsup*), which is unique to *Drosophila* and acts as a negative regulator of TH, likely in part through an interaction with VMAT and a coupling of DA synthesis to its transport (Stathakis et al., 1999; Wang et al., 2011).

**TABLE 1:** Genes involved in biosynthesis pathway of different monoamines in *C.elegans* and *Drosophila.*

Monoamine	Gene name	*Drosophila melanogaster*	*Caenorhabditis elegans*
Dopamine	Tyrosine hydroxylase (TH)	*pale*	*cat-2*
	Tetrahydrobiopterin (BH_4_)	*Punch*	—
	Aromatic amino acid decarboxylase (AADC)	*Ddc*	*bas-1*
	Vesicular monoamine transporter (VMAT)	*Vmat*	*cat-1*
	Catecholamines up	*Catsup*	—
	Dopamine Transporter (DAT)	*Fumin*	*dat-1*
Serotonin	Tryptophan hydroxylase (TPH)	*Trh*	*tph-1*
	DDC, also known as AADC	*Ddc*	*bas-1*
	Serotonin Transporter	*SERT*	*mod-5*
Tyramine	Tyrosine decarboxylase (TDC)	*Tdc*	*tdc-1*
Octopamine	Tyramine *ß* -hydroxylase	*Tbh*	*tbh-1*
Histamine	Histidine decarboxylase	*Hdc*	—
	Histidine transporter	*Tadr*	—
	Putative synaptic vesicular transporter	*lovit*	—
	Carcinine Transporter	*CarT*	—
	N-β-alanyl dopamine synthase	*ebony*	—
	β-alanyl histamine hydrolase	*tan*	—

DA synthesis is similarly conserved in *C. elegans*, (Figure 1, see above) (Sulston et al., 1975). The components include *C. elegans* TH, encoded by *cat-2*; and an aromatic L-amino acid decarboxylase (AADC) homolog that is encoded by *bas-1* (Lints and Emmons, 1999; Hare and Loer, 2004) (Table 1). The gene *cat-1*, a homolog of human VMAT, is expressed in all eight dopaminergic neurons in the hermaphrodite (Duerr et al., 1999). Like *Drosophila*, *C. elegans* has only one VMAT gene, in contrast to the two found in humans. Importantly, *C. elegans* VMAT is more akin to human VMAT2 in the sense that both have a higher affinity for histamine. Moreover, a major difference between the two human variants and the *C. elegans* protein is that worm VMAT, like that of *Drosophila*, shows a submicromolar affinity for DA (Duerr et al., 1999; Greer et al., 2005).

#### 2.1.2 Synaptic transmission

**FIGURE 2:**
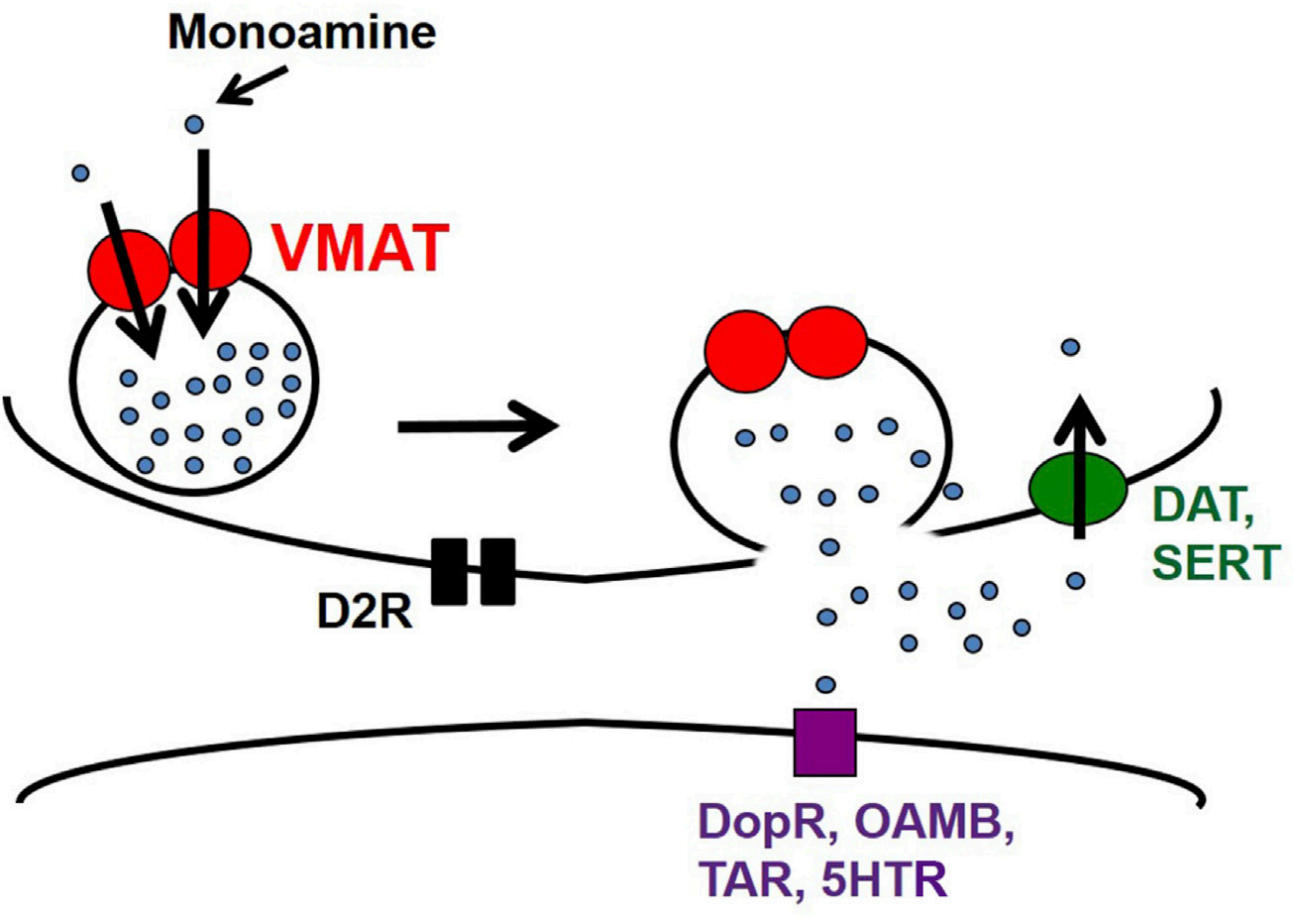
Synaptic release in a monoaminergic neuron. Following synthesis, dopamine, serotonin, octopamine, and tyramine are packaged into synaptic vesicles by VMAT for exocytosis. Excess monoamines are recycled whole *via* dopamine transporter (DAT) or serotonin transporter (SERT). Extracellular monoamines can also bind to postsynaptic receptors to relay the signal downstream.

DA is a highly potent neurotransmitter that is released mainly from non-ionotropic synapses (except in instances of co-release). Typically, it is synthesized in the cytosol (or recycled from the extracellular space) and packaged into synaptic vesicles for exocytotic release, after which it binds to post-synaptic DA receptors (usually but not exclusively D1-like) for subsequent relay of the signal downstream (see Figure 2). It is important to emphasize that synaptic vesicle release constitutes just one form of regulated release. Release through large-dense core vesicles (LDCVs) represents another form, with consequences for both regulation and modulation of behaviors in a circuit-specific way (Grygoruk et al., 2014). Moreover, there is evidence in *Drosophila* for non-vesicular release of DA through a DAT-mediated efflux in response to treatment with the psychostimulant amphetamine (Pizzo et al., 2013).

In *C. elegans*, DA is one of four biogenic amines which acts through its receptors to modulate important behaviors like locomotion in a manner similar to that seen in mammals (Chase and Koelle, 2007). An antagonistic relationship between DOP-3 (D2-like receptor, encoded by *dop-3*) and DOP-1 (D1-like receptor, encoded by *dop-1*) provides a mechanism through which extra-synaptic DA is regulated, with behavioral effects downstream through the recruitment of Gα_o_ and Gα_q_ (Chase et al., 2004).

To further elucidate protein interactions at the synapse, Fluorescence Recovery after Photobleaching (FRAP) studies have been done with *dop-2* and *dat-1* (the *C. elegans* homolog of human DAT) deletion mutants. Experiments have shown that excess DA causes worm immobilization, which can be observed through a swimming induced paralysis (SWIP) assay in which the greater the DA presence at the synapse, the faster the animals become paralyzed (McDonald et al., 2007). Since DAT-1 clears the synapse of DA, *dat-1* deletion mutants have excess synaptic DA, causing them to paralyze faster while swimming, compared to their wildtype counterparts (McDonald et al., 2007). DOP-2 is a known negative modulator of DA signaling and accordingly, *dop-2* mutants show higher rates of SWIP, which is indicative of elevated synaptic DA. The increased FRAP recovery in animals carrying a lesion in the *dop-2* gene indicates that these mutants lack a mechanism that downregulates release of DA (Formisano et al., 2020). In addition, the inability of the *dat-1* mutants to recycle DA into the cytosol may account for the increased synaptic DA, as measured by SWIP and FRAP studies (Formisano et al., 2022). However, it should be noted that those studies are indirect readouts of extracellular DA and direct quantifications of the neurotransmitter levels would be needed to consolidate the case for effects of DOP-2 and DAT-1 on synaptic DA release.

#### 2.1.3 Recycle and breakdown

Two critical events are essential in maintaining DA homeostasis. DA is recycled through DAT, which is encoded in *Drosophila* by *Fumin* (discovered as a sleep mutant (Kume et al., 2005)) and in *C. elegans* by *dat-1* (Carvelli et al., 2008). This action serves to reduce extracellular DA levels and its subsequent potentiation of synaptic activity. dDAT is a member of the solute carrier 6 (SLC6) family of transporters that reuptakes neurotransmitters across the plasma membrane by alternating between its outward and inward facing conformations coupled with Na^+^/Cl^−^ transport. Morphologically, DAT consists of 12 transmembrane spanning helices with multiple binding sites, including ion-binding, substrate binding, and cholesterol binding sites (Penmatsa et al., 2013). Unlike DA recycling, much less is known about its breakdown in *Drosophila*. This dearth of knowledge also contrasts with the depth of the literature on the subject in rodents and other mammalian models. In mammals, DA breakdown is catalyzed by monoamine oxidase (MAO), which converts DA to 3,4-dihydroxyphenylacetic acid (DOPAC) (Di Monte et al., 1996). MAOs are flavin-dependent enzymes that are physically tethered to the outer mitochondrial membrane. There are two forms that are differentially expressed within the CNS. MAO-A is found predominantly in neurons, while MAO-B is found in astrocytes (Alborghetti and Nicoletti, 2019). MAO-B is a target for anti-Parkinson’s disease therapeutics because of its ability to effect the breakdown of DA and thus regulate the available amount of that neurotransmitter (Oertel and Schulz, 2016). *Drosophila* has no known MAO gene, although DOPAC has been detected in fly head extracts and its levels are sensitive to oxidative stress, suggesting that a functional homolog of MAO may exist in the organism (Wang et al., 2011). Besides DA breakdown, another mechanism of its removal is its conjugation with *ß*-alanine by *ß*-alanine-dopamine synthase, which is encoded by *ebony* in *Drosophila* (Wright, 1987; Borycz et al., 2002) to form melanin.

An interesting facet about *C. elegans* DAT is that it likely executes its function through an interaction with DOP-2 (Formisano et al., 2020) (*Synaptic transmission* above). Moreover, there are suggestions that the *C. elegans* SNARE protein, syntaxin 1A homologue, UNC-64 represses DAT (Carvelli et al., 2008), although the underlying mechanism is not fully understood. The breakdown of DA (and other monoamines) has not been well characterized in *C. elegans*, even though the nematode contains homologs of human catabolic enzymes. These include MAO and catechol-o-methyltransferase (COMT), responsible for converting DA to DOPAC or to 3-methoxytyramine (3-MT), respectively. Of the proteins that bear some homology to MAO, AMX-1, AMX-2, and AMX-3 each have 35–40% amino acid similarity with MAO-A and MAO-B. AMX-2 has an α helical domain similar to the C-terminal domain in MAO-A (Wang et al., 2017). When wildtype worms were exposed to a MAO-B inhibitor, DA levels were increased. The same results were seen when COMT inhibitor was applied (Schumacher et al., 2015). Both studies suggest that an MAO-like mechanism exists for the degradation of DA.

### 2.2 DA receptors

DA receptors play key roles in DA signal transduction, modulation, and cell-cell communication. Mammals possess five types (D1-D5) of DA receptors, which decades ago were classified into D1-like and D2-like groups based on their physiology and mechanism of action (Kebabian and Calne, 1979). Both groups of DA receptors represent 7-transmembrane, rhodopsin-like (Class A) G-protein coupled receptors (GPCR) (Missale et al., 1998). The D1-like receptor group is composed of D1 and D5 receptor subtypes and it is known for coupling to adenylate cyclase stimulatory Gα protein resulting in an increase of cyclic AMP (cAMP) production. Typically, stimulation of D1-like receptors positively influences excitability of the affected cell and leads to an increase in NMDA receptors (Surmeier et al., 2010). Like its mammalian cognates, fly D1-like receptors facilitate an increase in the levels of cAMP upon DA binding (Sugamori et al., 1995). Moreover, there are two receptors that are classified as D1-like in *Drosophila* (Dop1R1 and Dop1R2, also known as DUMB and DAMB, respectively). The D2-like receptor group includes D2, D3, and D4 receptor subtypes, which upon agonist binding generally inhibit adenylyl cyclase through coupling to Gα_i_/Gα_o_ subunits, and further inhibit cAMP production (Neve et al., 2004). Dop2R (aka DD2R) is the only D2-like receptor in flies. Another aspect of DA receptor conservation between flies and human DA receptors is their desensitization and internalization by the action of GPCR kinases (GRKs) and arrestin proteins (Karam et al., 2020). Upon agonist binding, GPCR may bind one or more GRKs, resulting in phosphorylation of its residues at the specific termini. The phosphorylated GPCR serves as a binding site for *ß*-arrestins, causing desensitization of the GPCR and further termination of receptor signaling (Kohout and Lefkowitz, 2003). D2 receptors have known auto-receptor function, exemplified both by their presence in DA neurons and the ample demonstration of that function in mammalian models (Ford, 2014).

DA receptor function is similarly conserved in *C. elegans* with DA receptors characterized as either D1-like or D2-like receptors (reviewed in (Chase and Koelle, 2007)). Three of the four DA receptors found in *C. elegans* are functionally similar to their mammalian counterparts. DOP-1 (encoded by *dop-1*) is classified as a D1-like receptor while DOP-2 and DOP-3 are D2-like receptors (Chase and Koelle, 2007). DOP-4 (encoded by *dop-4*) is a D1-like receptor that is unique to invertebrates (Sugiura et al., 2005; Pandey and Harbinder, 2012). Research in *C. elegans* has led to additional key findings regarding DOP-2 function and its interacting partners (see *Synaptic Transmission* above). A case in point is the mediation of synaptic vesicle fusion by DOP-2 and the presynaptic acid sensing Na^+^ ion channel (ASIC-1), encoded by *asic-1*. FRAP studies on *asic-1* and *dop-2* deletion mutants have shown that when measuring recovery rates, vesicular fusion was inhibited in *asic-1* mutants, but quickly recovered in *dop-2* mutants. We note that it is not clear whether the ASIC-1 effect on synaptic release is direct or indirect, but this is an important question that merits further inquiry. These results and others suggest that DOP-2 may physically associate with the inhibitory unit of Gα_i_ of GPA-14 (Pandey and Harbinder, 2012; Formisano et al., 2020). Moreover, ASIC-1, DAT-1 and DOP-2 appear to act in concert to modulate synaptic DA levels (Formisano et al., 2020), with ASIC-1 proposed as working to generate a positive feedback loop during that action (Voglis and Tavernarakis, 2008).

### 2.3 The D2 autoreceptor

In mammals, D2-autoreceptors play a crucial role in synthesis, release, and reuptake of dopamine (Ford, 2014). Their activation usually diminishes the dopamine levels by acting on intracellular calcium signaling, and thus considered inhibitory (Benoit-Marand et al., 2001). While the vast majority of D2-like receptors are localized to non-dopaminergic neurons, the D2-autoreceptors are found solely on dopamine neurons, more specifically somatodendritic and axon terminal regions (Missale et al., 1998). Pharmacological studies in fruit flies have found that *Drosophila* D2 receptors function as autoreceptors and regulate dopamine release (Vickrey and Venton, 2011) and may play a role in the rescue of DA neurodegeneration induced by MPP+ (Wiemerslage et al., 2013). In Vickrey and Venton (2011), D2 receptor agonists, bromocriptine and quinpirole, decreased optogenetically stimulated dopamine release, while the antagonists, flupenthixol, butaclamol, and haloperidol, had an opposite effect. In *C. elegans* the D2-like receptor DOP-2 (encoded by *dop-2*) is expressed on dopaminergic neurons (Suo et al., 2003). Pandey et al. (2021) further demonstrated a modulatory role for DOP-2 in ethanol-dependent behaviors by exposing *dop-2* mutants to ethanol, resulting in *dop-2* mutant worms moving with an Ethanol Induced Sedative behavior characterized in part by decreased motion in the posterior end of the worm, in contrast to wildtype worms subjected to the same treatment. FRAP conducted at the PDE synapse showed excess DA in these mutants. Moreover, the constitutive fusion of DA-carrying vesicles at the dopaminergic synaptic termini has also been found to be regulated by DOP-2 *via* a negative feedback loop (Formisano et al., 2020).

### 2.4 DA and behavior

#### 2.4.1 DA-mediated behavior in *Drosophila*

Consistent with its status as a major excitatory neurotransmitter, DA plays an important role in the regulation of several behaviors and physiological processes. Much of its action is facilitated by DA neurons, which innervate the fly brain and are arranged in distinct clusters (Mao and Davis, 2009). Moreover, DA functions through all stages, from embryonic development through adulthood (Monastirioti, 1999; Hsouna et al., 2007). In adults, its range of activity includes the regulation of sleep, learning and memory, and arousal (Lebestky et al., 2009; Chen et al., 2013; Pizzo et al., 2013; Nall and Sehgal, 2014). A selection of behaviors regulated or modulated by DA is described below.

##### 2.4.1.1 Locomotion

The regulation of locomotion is complex and involves many neurotransmitter systems including DA, OA, and 5-HT. OA is the predominant excitatory neurotransmitter in the fly locomotion system while DA plays a more modulatory role in how the behavior is maintained (Pizzo et al., 2013). Even so, there are at least two locomotion circuits—the baseline locomotion and the touch response circuits (Simon et al., 2009), and it is possible that DA plays differential roles in both. In *Drosophila* larvae, *VMAT* mutants show reduced locomotion and diminished electrical activity in motor neurons within the neuromuscular junction (NMJ) (Simon et al., 2009; Chen et al., 2013), although the contribution of DA relative to OA was not parsed in the study.

##### 2.4.1.2 Courtship and male fertility

DA has long been known to play an important role in regulating courtship, as males with DA levels depleted by 3-iodo tyrosine show defects in the performance of that behavior, an effect that is rescued by an introduction of a wildtype TH transgene (Neckameyer, 1998). The circuitry underlying this function has been investigated (Kuo et al., 2015) and an involvement of DA receptors has been reported (Lim et al., 2017). Male fertility is similarly DA-dependent, as infertile male *DVMAT* null mutants can be partially-rescued with DA neuron-specific expression of TH (Chen et al., 2013). Importantly, a dopaminergic role in courtship and male fertility is functionally-redundant with OA (Chen et al., 2013). Moreover, Chen et al. show that severe fertility and courtship defects in *dVMAT* null mutants can be rescued through a DA or OA neuron specific expression of VMAT, but neither one alone provides full rescue. Although the precise nature of this redundancy is not fully understood, it suggests an interaction between the two circuits in regulating male fertility.

##### 2.4.1.3 Egg-laying and female fertility

*Drosophila* females exhibit a specific behavior of selecting the most suitable site for their egg deposition, which involves a simple decision-making process (Wu et al., 2015). In many instances, oviposition takes place in fermenting fruits which are rich in ethanol. Flies test the concentration of the metabolite in the food source and then decide if the environment is favorable for progeny survival in a DA-dependent process. The data show that dopaminergic neurons relay the gathered sensory inputs about ethanol concentration to the mushroom body (MB) and the ellipsoid body (EB), which are further transmitted by MB α’β’ neurons and EB R2 neurons. A 5% ethanol concentration has been shown to activate the protocerebral anterior medial (PAM) and the protocerebral posterior medial (PPM3) neurons, whereas protocerebral posterior lateral (PPL1) neurons appear to inhibit MB α’β’ neurons, resulting in reduced oviposition preference (Azanchi et al., 2013). Importantly, another study demonstrated that activity in MB αβ neurons is required for egg-laying behavior (Wu et al., 2015). Female fertility is also dependent on DA, although this effect appears modulatory compared to a more dominant role by OA (Chen et al., 2013).

##### 2.4.1.4 Learning and memory

In *Drosophila,* the MB is the major site for associative learning (Busto et al., 2010; Aso et al., 2014). And although other neurotransmitters are involved in different aspects of information acquisition, DA has long been known to play a key role in aversive learning (Schwaerzel et al., 2003; Schroll et al., 2006). Fascinating new knowledge regarding the function of DA in this behavior has been uncovered over at least the past decade. One of which is that in addition to its role in aversive learning, DA also controls appetitive learning (Kim et al., 2007). Moreover, DA, through the action of approximately two DA neurons, plays a key role in forgetting, the process through which memory is lost (Berry et al., 2018; Sabandal et al., 2021). Intriguingly, the circuitry of transient forgetting, i.e., temporary impairment of memory retrieval, has been mapped to a single DA neuron (Sabandal et al., 2021). DA’s role in cognition involves DA receptor DAMB signaling through the Gα_q_ to stimulate cAMP (Himmelreich et al., 2017). DA neurons (DANs) of the PPL1 cluster project axons into the MB and form a key component of that cognitive process (Aso et al., 2014). The modulatory activities of these neurons mediate both learning and the rate of forgetting aversive memories (Berry et al., 2018). Importantly, the dynamic interaction between DA and OA seen in other behavioral circuits (see above) also appears to be at play here. In this case, OA mediates appetitive cognitive performance and is responsible for providing that appetitive information input into the MBs (Schwaerzel et al., 2003; Busto et al., 2010; Nall and Sehgal, 2014).

#### 2.4.2 DA-mediated behavior in *C. elegans*

##### 2.4.2.1 Adaptation

Adaptation is among many worm behaviors regulated by DA. Research using mutational analysis has shown that DA signaling modulates non-associative learning in aversive conditions in *C. elegans via* the D1-like receptor, DOP-4 (Ardiel et al., 2016). When exposed to an olfactory stimulus, worms normally exhibit adaptation following the exposure, but after odor presentation, *cat-1* and *cat-2* mutant worms are defective in dopaminergic neuron signaling and thus impaired in adaptation, indicating that DA functions in state-dependent adaptation in *C. elegans* (Bettinger and McIntire, 2004).

##### 2.4.2.2 Locomotion and mating related behavior

Ordinary sinusoidal movements of the worm and several essential life processes including egg-laying and locomotion have been shown to be coordinated by DA (Cermak et al., 2020). Further, the neurotransmitter is responsible for increased movement in males while searching for mates; as well as repression in hermaphrodites which rely on self-fertilization (Suo et al., 2019). A key study on DA receptors delineated an antagonistic relationship in the regulation of locomotion between the D2-like receptor, DOP-3, and the D1-like receptor, DOP-1, such that a knockout of the former led to locomotion impairments that were reversed by a knockout of the latter. Importantly, this group determined that extrasynaptic DA was key to the regulatory action of both receptors (Chase et al., 2004). Moreover, changes in behavior related to hunger and starvation are modulated by AIY and RIG interneurons which are also controlled by DA (Rengarajan et al., 2019).

##### 2.4.2.3 Learning

The mechanisms through which DA mediates these essential activities are not fully understood but it is likely that some of the regulation occurs through an interaction between the adaptor protein GPA-14 and DOP-2 (Mersha et al., 2013). Studies conducted with *gpa-14* and *dop-2* mutants as well as with the double mutant *gpa-14;dop-2* show disparities in non-associative and associative learning. During a non-associative learning assay, both single and double deletion mutants learned faster as compared with controls (Mersha et al., 2013). Importantly, this effect was eliminated with exposure to exogenous DA. Conversely, associative learning experiments strengthened the theory of an interaction between Gα subunit adaptor protein GPA-14 and DOP-2, as both single deletion mutants and double deletion mutants showed similar decreases in associative learning when compared with controls (Mersha et al., 2013).

##### 2.4.2.4 A dopaminergic role during foraging

Basal slowing or basal slowing response (BSR), a behavior exhibited by worms in the presence of its food, bacteria, is another activity that is modulated by DA (Sawin et al., 2000; Rodríguez-Ramos et al., 2017). One key feature is that a DA-mediated neural circuit appears to sense specific properties of the bacteria and allows for a slowing of the worm’s movement to maximize the time spent on food (Sawin et al., 2000). Also related to basal slowing, and mediated in part by DA, is an area-restricted search (ARS) behavior in which DA and glutamate mediate a time-dependent regulation of turning frequency to maximize time spent in areas of food abundance during resource scarcity (Hills et al., 2004). Intriguingly, *C. elegans* have a well-developed circuit for sensing variability in their food environment and an ability to modify their behavior accordingly (Calhoun et al., 2014). This latter point is worth emphasizing because it forms a basis through which sensory experience can be predictive of subsequent local foraging behavior.

## 3 Serotonin

### 3.1 5-HT metabolism and regulation

#### 3.1.1 Biosynthesis, transport, release, recycle, and breakdown

**FIGURE 3:**
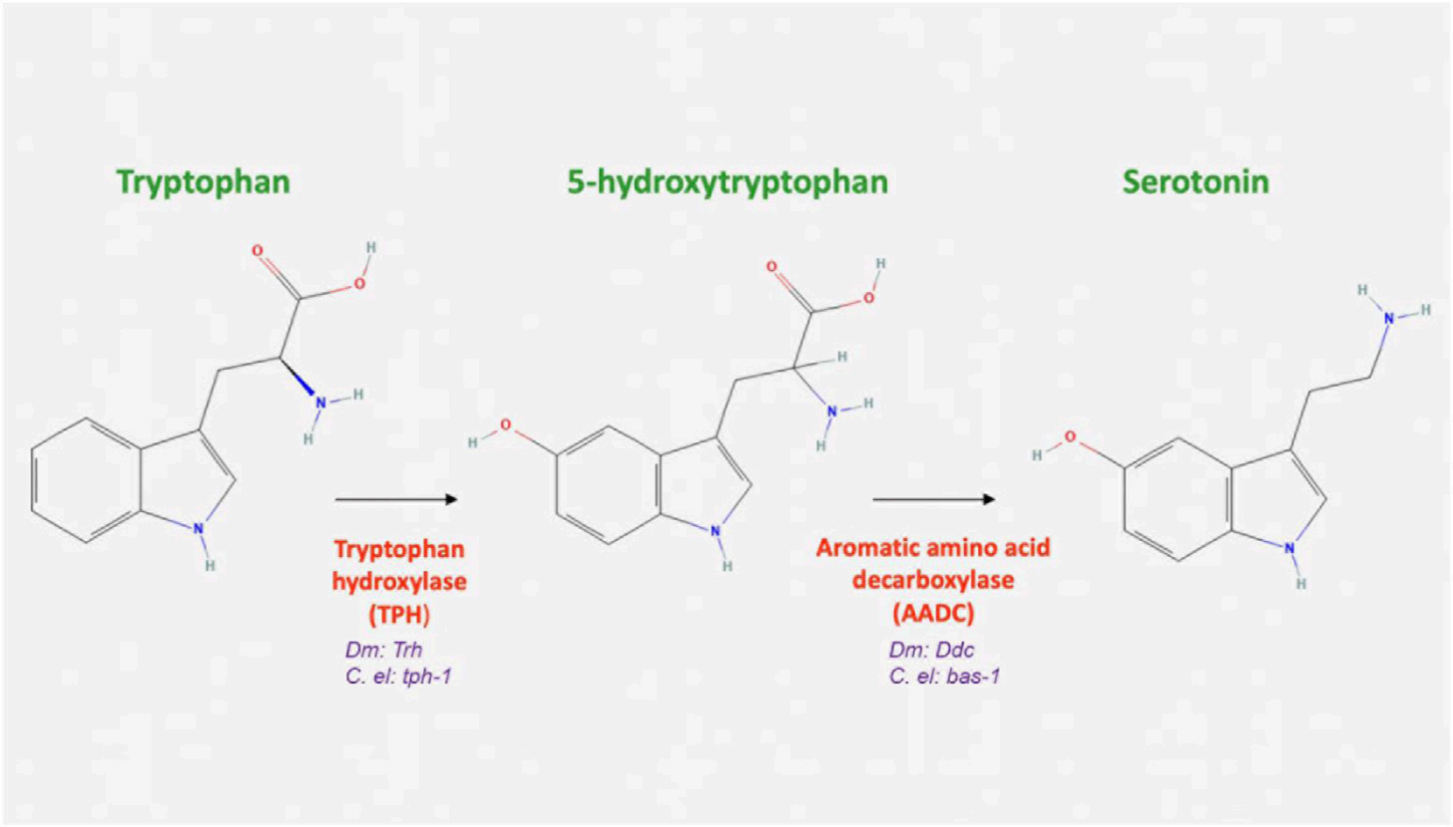
Serotonin biosynthesis. Serotonin is synthesized in the cytosol from the amino acid tryptophan. It is first converted to 5-hydroxytryptophan hydroxylase, encoded in *Drosophila* by *Tph* and in *C. elegans* by *tph-1*, a first and rate-limiting enzyme in the pathway. This substrate is then decarboxylated into serotonin by the aromatic acid decarboxylase, encoded by *Ddc* in *Drosophila* and *bas-1* in *C. elegans* (All chemical structures were retrieved and modified from Pubchem; https://pubchem.ncbi.nlm.nih.gov/).

The 5-HT biosynthetic pathway is conserved among flies, worms, and humans. In *Drosophila* and *C. elegans,* the first step in 5-HT synthesis is the rate-limiting hydroxylation of the amino acid tryptophan, which generates 5-hydroxytryptophan and is catalyzed by tryptophan hydroxylase (TPH) (Figure 3; Table 1). In *Drosophila*, DTPH has been shown to function dually, hydroxylating both phenylalanine and tryptophan (Coleman and Neckameyer, 2005). In worms, this enzyme is encoded by *tph-1* (Table 1), and is conserved, sharing 61% amino acid identity in the catalytic domain with its mammalian cognate (Sze et al., 2000). Its activity is based solely on the substrate exhibiting its differential hydroxylase activity. When DTPH uses phenylalanine as a substrate, regulatory control is observed through catecholamine inhibition or decreased TPH activity. When tryptophan is used as a substrate, the aforementioned regulatory mechanism is not observed, indicating the possibility of phenylalanine’s hydroxylation by DTPH as its primary role (Coleman and Neckameyer, 2004; 2005). Additionally, another enzyme, tryptophan hydroxylase (DTRH), has extensive homology to mammalian TPH and is expressed in neurons (Neckameyer et al., 2007). DDC (known as AADC in *C. elegans* is encoded by *bas-1* (Hare and Loer, 2004)) catalyzes the final step in 5-HT synthesis, the decarboxylation of 5-hydroxytryptophan to 5-HT. After synthesis, 5-HT is packaged into synaptic vesicles by VMAT for exocytotic release, as has been demonstrated using pharmacological agents that target 5-HT synthesis and packaging (Borue et al., 2009). In worms, flies, and mammals, VMAT localizes to synaptic vesicles in serotonergic neurons (Duerr et al., 1999; Chen et al., 2013; Schafer et al., 2013).

Like DA, 5-HT is recycled into the presynaptic neuron in its entirety. The protein that mediates that effect is the 5-HT Transporter (SERT), making it an essential component of 5-HT regulation (Pham et al., 2020). dSERT (*Drosophila* ortholog of SERT) takes up excess 5-HT from the synaptic cleft (Giang et al., 2011) and is expressed in serotonergic neurons of larvae and adult fruit flies (Monastirioti, 1999; Giang et al., 2011). The decreased SERT activity has been linked to the development of depressive states and anxiety, such that Selective Serotonin Reuptake Inhibitors have been widely used for the treatment of depression (Borue et al., 2010). These compounds increase the levels of 5-HT by inhibiting its reuptake into the presynaptic terminus. Moreover, in studies with channelrhodopsin2-mediated release of 5-HT, pharmacological blockade of SERT with cocaine and fluoxetine led to sustained 5-HT signaling, demonstrating a role for the transporter in 5-HT clearance from the extracellular space (Borue et al., 2009). This use of fast-scan cyclic voltammetry to directly measure monoamine release at larval synapses was an important advance that has since been performed by others (Majdi et al., 2015). 5-HT can also be taken up by DAT, but at an order of magnitude less than by SERT, making it unlikely that DAT plays a significant role in 5-HT clearance (Borue et al., 2009).

5-HT plays many key roles in *C. elegans*, including sensing and ingesting food, locomotion, metabolism, learning, and egg-laying (Horvitz et al., 1982; Weinshenker et al., 1995; Sze et al., 2000; Hukema et al., 2008). Currently, seven serotonergic neurons have been identified in both male and hermaphrodite worms and can be found in the head. These include two neurosecretory motor neurons (NSM), one ring interneuron (RIH), a pair of amphid sensory neurons (ADF) and two ring interneurons (AIM) (Horvitz et al., 1982; Loer and Rand, 2022). Beyond these neurons, at least seventeen more are specific to the male worm and at least four more are found in the hermaphrodite. These have been thoroughly reviewed on Wormatlas (Loer and Rand, 2022). Interestingly, the Neurosecretory Motor (NSM) neuron pair synthesizes the largest amount of 5-HT, but does not have any postsynaptic connections (Loer and Rand, 2022). As in flies, 5-HT is similarly synthesized in *C. elegans* from tryptophan (Figure 3) (tryptophan hydroxylase is encoded by *tph-1;* AADC is encoded by *bas-1* (Sze et al., 2000; Hare and Loer, 2004). Moreover, like in flies, and mammals, VMAT localizes to synaptic vesicles in serotonergic neurons (Duerr et al., 1999). SERT (encoded by *mod-5*) was identified about two decades ago as the only *C. elegans* reuptake transporter (Ranganathan et al., 2001), and like in flies, recycles 5-HT into the presynaptic neuron. MAO genes have been identified in *C. elegans*, and are a likely candidate for 5-HT breakdown (Hobert, 2013). However, little has been done to characterize these genes and much remains poorly understood regarding how 5-HT is degraded in *C. elegans*.

### 3.2 5-HT receptors

Fruit flies possess five serotonergic receptors: 5-HT1A, 5-HT1B, 5-HT2A, 5-HT2B, and 5-HT7. All of them belong to the GPCR family, and like in humans, they have distinct effects upon activation. Receptors in the first family, 5-HT1A and 5-HT1B, act through the Gα_i_ pathway to inhibit the generation of cAMP, whereas 5-HT7, the only member of the 5-HT7 family in *Drosophila,* stimulates the production of cAMP. 5-HT2A and 5-HT2B act through the PLC-IP_3_ signaling pathway to increase intracellular calcium. 5-HT1A and 5-HT2A receptors negatively regulate attraction to high acetic acid levels (He et al., 2020). Conversely, 5-HT1B positively regulates attraction to low concentrations of acetic acid but negatively so to high concentrations of the odorant (He et al., 2020). An intriguing role for 5-HT receptors has also been described in the modulation of depression-like states in flies, defined as voluntary reduced walking ability to avoid punishment or learned helplessness (Batsching et al., 2016), due to reduced 5-HT released in the MB (Ries et al., 2017). The decrease in 5-HT levels in flies is restored by feeding the 5-HT precursor 5-hydroxy-L-tryptophan, which provides a sense of relief that is mediated by 5-HT1A receptors in the α/β-lobes of the MB, whereas 5-HT1B receptors control behavioral inactivity in γ-lobes (Ries et al., 2017). 5-HT receptors are also involved in other behaviors and physiological outputs (see section 3.2). These include the entrainment of the *Drosophila* circadian clock to light (Yuan et al., 2005) and aggression (Alekseyenko et al., 2014).

Much of the understanding regarding the function of mammalian 5-HT receptors has come from pharmacological analyses, which has helped establish the functional interactions that occur among receptor subtypes 5-HT1A and 5-HT2; as well as between 5-HT2 and 5-HT7. These analyses aid in understanding how these circuits mediate short-term learning and memory. For instance, the examination of long-term memory revealed that 5-HT1A, 5-HT2, and 5-HT7 are important for consolidation and recall (Johnson et al., 2011). Note that care must be taken to extrapolate findings from mammalian work to flies, since the pharmacology of the receptors in flies may be different.

At least four types of G-protein serotonergic receptors have been identified in *C. elegans—*SER-1 (encoded by *ser-1*), SER-4 (encoded by *ser-4*), SER-5 (encoded by *ser-5*), and SER-7 (encoded by *ser-7*) (Hamdan et al., 1999; Hobson et al., 2003; Hapiak et al., 2009; Harris et al., 2009). Additionally, another receptor, MOD-1 (encoded by *mod-1*), a 5-HT-gated chloride channel (Ranganathan et al., 2000), has been found. At least three of the five 5-HT receptor types are expressed in pharyngeal neurons or muscles involved in food processing. SER-1, SER-4, and SER-7 all play a role in the modulation of pharyngeal pumping rate (Lee et al., 2017). SER-5 is involved in the regulation of aversive behaviors and egg-laying (Hapiak et al., 2009; Harris et al., 2009). Moreover, the slowing behavior upon encountering food is caused by the extrasynaptic 5-HT release from the NSM and ADF neurons, and involves SER-4 and MOD-1, which are found throughout the body on interneurons and motor neurons (Gürel et al., 2012). This experience-dependent behavior is also modulated by SERT (encoded by *mod-5*) (Ranganathan et al., 2001).

### 3.3 5-HT and behavior

#### 3.3.1 Drosophila

The adult brain of *Drosophila* is composed of approximately 80 serotonergic neurons, distributed in a spatially diverse pattern of clusters in the CNS (Monastirioti, 1999), which are involved in modulation of sleep, feeding, aggression, synapse formation, and long-term memory formation (Yuan et al., 2005; Alekseyenko et al., 2014; Scheunemann et al., 2018).

##### 3.3.1.1 Locomotion

5-HT mediates walking in *Drosophila*. The activation of serotonergic neurons located in the ventral nerve cord (VNC) slows walking speed, whereas inhibition of the same serotonergic neurons enhances it (Howard et al., 2019). 5-HT plays a more subtle role in the startle response circuit, as decreased speed to re-acclimate after an initial startle in *dVMAT* mutants can be rescued with a serotonergic neuron-specific rescue of VMAT (Chen et al., 2013).

##### 3.3.1.2 Learning and memory

An essential role for 5-HT in olfactory learning and memory was first demonstrated more than a decade ago with pharmacological and genetic approaches that targeted classes of 5-HT receptors (Johnson et al., 2011). Work since then has established that the 5-HT7 receptor, located in the *Drosophila* MB neurons, regulates appetitive olfactory learning in larvae in a cAMP-dependent manner such that the down-regulating d5-HT7 receptor expression, specifically in the MB neurons, resulted in impaired olfactory appetitive learning (Ganguly et al., 2020). The role of 5-HT contrasts with that of DA in olfactory learning in adults, in which DA is involved in both appetitive and aversive learning (Berry et al., 2018).

##### 3.3.1.3 Rest and sleep

In *Drosophila*, sleep has been described as an increased threshold for arousal, and with respect to the circadian clock, it is regulated independently (Beckwith and French, 2019). Despite this autonomy, the consequential relationship between circadian rhythm and sleep cannot be overemphasized. Just as important has been the role of *Drosophila* in elucidating the molecular mechanisms underlying circadian rhythm and sleep since the discovery of *period* in 1971 in the laboratory of Seymour Benzer (Konopka and Benzer, 1971), *timeless* (Sehgal et al., 1994), and *Clock* (Allada et al., 1998) in flies (for review, see (Dubowy and Sehgal, 2017)). The regulation of rest in *Drosophila* correlates strongly with, and is subject to similar molecular regulations as sleep in mammals (Shaw et al., 2000). Along those lines, molecular markers modulated by sleep and waking have been identified, including cytochrome C oxidase, whose role is conserved in flies and mammals. Importantly, 5-HT is known as a baseline sleep-promoting neurotransmitter and sleep homeostasis modulator in flies (for review see (Nall and Sehgal, 2014)). 5-HT also regulates the entrainment of circadian behavioral rhythms in the fruit fly by influencing the molecular response to light. Specifically, the entrainment mechanism is inhibited by 5-HT through the 5-HT1B receptor (Yuan et al., 2005). Moreover, a sleep study Yuan et al. (2006) identified d5-HT1A receptor as a key sleep-regulating factor. Flies with genetically altered d5-HT1A receptor exhibited short and fragmented sleep, while transgenic expression of the receptor in MBs rescued the sleep reduction phenotype. Additionally, the loss of 5-HT2B receptor has been found to negatively affect both sleep time and sleep rebound after sleep deprivation; and that receptor localization to the dorsal fan-shaped body (dFB) has been characterized as essential for sleep homeostasis (Qian et al., 2017). Further advances have been made in uncovering the neural circuitry that links sleep to memory consolidation. The Griffith lab has reported that a single pair of serotonergic neurons, which are essential for memory consolidation are also sleep-promoting cells (Haynes et al., 2015). Intriguingly, there is a different dimension to the relationship between sleep and cognition, such that a serotonergic circuitry that controls sleep bouts also mediates cognitive function (Liu et al., 2019).

##### 3.3.1.4 Social attraction

Exciting recent research has uncovered a clear role for 5-HT in social attraction. Serotonergic neurons within ɣ lobe of the MB are activated in response to social experience (Sun et al., 2020). Indeed, RNAi knockdown of 5-HT1B, specifically in the Kenyon Cells ɣ neurons, caused reduced affinity for social spaces, indicating the importance of 5-HT and its 5-HT1B receptor for social motivation and sociability in *Drosophila* (Sun et al., 2020).

##### 3.3.1.5 Olfaction

5-HT is known to modulate olfactory processing in the fly antennal lobe by enhancing the olfactory responses of projection neurons (Dacks et al., 2009). Furthermore, recent work has shown distinct contributions of the somatodendritic 5-HT transporter in *Drosophila* olfaction. Moreover, Xu et al. (2016) found 5-HT to be involved in ethanol attraction, as flies with pharmacologically and genetically increased 5-HT levels exhibited significant decrease in preference for ethanol’s scent and ethanol attraction. *Drosophila* developed ethanol tolerance after a single and multiple exposures to the alcohol in a dose-dependent manner (Scholz et al., 2000). Importantly, when comparing wild type and a SERT R599A C-terminus mutant, which enriches SERT in somatodendritic compartments, were re-expressed in serotonergic neurons, only wildtype animals showed restored ethanol preference. However, ethanol preference was restored by the SERT mutant after its targeted expression in a specific set of deuterocerebral interneurons. WT SERT expression by contrast led to ethanol aversion. Together, the findings demonstrate roles for somatodendritic vs. axonal 5-HT transport in ethanol aversion (Kasture et al., 2019).

##### 3.3.1.6 Aggression

This behavior represents one of the best characterized effects of neuronal serotonergic signaling (Chen et al., 2002). The Kravitz group are among the pioneers in developing a fly model for aggression. Their work characterized numerous behavioral patterns, laying the foundation for further genetic dissection of aggressive behavior in flies. Importantly, research from the group showed that an inactivation of the entire serotonergic system resulted in flies that exhibited less aggressive behavior, whereas the induced activation of serotonergic neurons yielded flies that fought more (Alekseyenko et al., 2014). Additionally, a pair of serotonergic posterior lateral protocerebrum (PLP) neurons has been identified and implicated in the escalation of aggression. The silencing of these neurons reduced aggression in males, whereas their activation promoted aggressive behaviors (Alekseyenko et al., 2014), suggesting that 5-HT-PLP neurons interact with 5-HT1A receptors, which when activated reduce aggression.

#### 3.3.2 C. elegans

##### 3.3.2.1 Feeding

As noted above, 5-HT receptors are integral to both fast pumping (to obtain food) and slowing when worms encounter the food (Dernovici et al., 2007; Gürel et al., 2012). Importantly, 5-HT also regulates pharyngeal pumping, mediated through SER-4, which works with SER-1 to promote burst of pumping activity, and with SER-7 helps initiate feeding behavior in response to 5-HT (Lee et al., 2017).

##### 3.3.2.2 Enhanced slowing by food-deprived animals

Enhanced slowing response (ESR) can be described as a complimentary behavior to basal slowing. It involves food-deprived animals (as opposed to sated organisms that exhibit basal slowing) exhibiting an enhanced slowing response that allows them to spend a maximum amount of time foraging on bacteria, their food source, and it is regulated by a 5-HT neural circuit (Sawin et al., 2000; Rodríguez-Ramos et al., 2017).

##### 3.3.2.3 Egg-laying and fertility

Binding of 5-HT to a GPCR coupled to Gα_q_ homolog *egl-30*, activates vulval muscles, whereas binding to Gα_o_ homolog *goa-1* does the opposite (Shyn et al., 2003). Fernandez et al. (2020) outlined four serotonergic receptors and their role in egg-laying. SER-1 and SER-7 receptors stimulate egg-laying by coupling to Gα_q_ and Gα_s_, respectively, whereas SER-4 receptor inhibits egg-laying by coupling to Gα_o_. *ser-1, ser-5* and *ser-7* are expressed in HSN egg-laying neurons, suggesting that 5-HT from the HSN could act in a feedback loop to maintain HSN activity. In addition, serotonergic HSN neurons modulate the egg-laying circuit by innervating vulval muscles (Trent et al., 1983). The Koelle Lab also found that ERG potassium channel UNC-103 enables alternating between an inactive phase and a 5-HT-triggered active phase by limiting the excitability of egg-laying muscles (Collins and Koelle, 2013).

##### 3.3.2.4 Learning

5-HT plays an important role in learning. Worms are able to learn/associate a particular smell with the absence or presence of food, in a pathway that is mediated by 5-HT (Nuttley et al., 2002). Additional work by the Jansen lab showed that nematodes deficient in 5-HT showed increased chemotaxis, a form of associative learning, suggesting that 5-HT inhibits that behavior (Hukema et al., 2008).

##### 3.3.2.5 Locomotion

5-HT mediates several locomotor behaviors in *C. elegans*, including food-induced slowing and foraging (Dernovici et al., 2007) (also noted above). In a large-scale screen to identify genes that affect 5-HT-mediated slowing behavior in worms, genes encoding proteins MOD-1 and SER-4 were identified as candidates. Intriguingly, the two aforementioned genes are nearly completely non-overlapping in their expression pattern and act independently to regulate locomotion (Gürel et al., 2012). Additionally, it has been proposed that Protein Kinase G orthologs *egl-4* and *pkg-2* mediate 5-HT-induced paralysis in *C. elegans* (Olson and Koelle, 2019).

##### 3.3.2.6 Aversion

5-HT is involved in the modulation of aversive olfactory response in *C. elegans.* A previous fascinating study demonstrated that after prior exposure to pathogenic bacteria, *C. elegans* avoided odors from that strain, a response that required 5-HT release from serotonergic ADF neurons, and was mediated through MOD-1 receptors (see section 2.2 above) (Zhang et al., 2005).

5-HT is also known to modulate aversive response to octanol, a behavior that although dependent on glutamate, is modulated by 5-HT when released on the ASH nociceptive neuron (Chao et al., 2004). This work also showed that increased sensitivity to octanol can be achieved when animals are placed on food. Other groups have investigated the involvement of 5-HT receptors in mediating responses to octanol. One of them reported that three 5-HT receptors, SER-1, SER-5, and MOD-1 were all imperative for dilute octanol responses in the specific condition of aversion while on food (Harris et al., 2009). Moreover, placement of these receptors on neurons, such as the AIB and RIA interneurons, led to an interaction with ASH neurons, helping to combine sensory and motor signaling, and modulate the 5-HT sensitivity to octanol (Harris et al., 2009).

## 4 Tyramine

**FIGURE 4:**
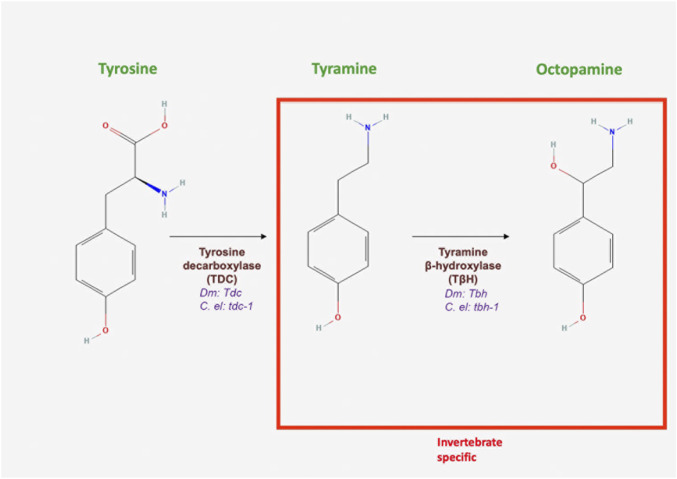
Octopamine and tyramine biosynthesis. Both neurotransmitters are synthesized in the same biochemical pathway. Tyrosine is decarboxylated into tyramine by tyrosine decarboxylase, encoded by *Tdc* in *Drosophila* and *tdc-1* in *C. elegans* (All chemical structures were retrieved and modified from Pubchem; https://pubchem.ncbi.nlm.nih.gov/).

In initial studies in locusts, TA distribution and uptake were already noted as independent of OA (Downer et al., 1993). Further advances have established physiological roles of TA independent of OA in invertebrate models (Donini and Lange, 2004; Saraswati et al., 2004; Cazzamali et al., 2005; Bayliss et al., 2013). Importantly, Nagaya et al. presented TA as a neuromodulator in *Drosophila* about two decades ago (Nagaya et al., 2002). Additional studies since then have identified a family of mammalian GPCRs as receptors for trace amines, two of which are homologous to *Drosophila* TyrRs (Borowsky et al., 2001). Although TA serves as a precursor for OA (Figure 4), there are tyraminergic neurons and behaviors controlled by TA alone. And it took some time before TA was recognized as an independent neurotransmitter (Alkema et al., 2005). Although both are now described as separate neurotransmitters, there remain a number of significant similarities. Moreover, some receptors like TYRA-2, determined to be either for OA or TA, show affinity for both neurotransmitters. In *C. elegans*, RIM neurons serve as both interneurons and motor neurons for muscles of the head and are distinguished as a TAergic neurons because they lack the enzyme tyramine *ß*-hydroxylase (encoded by *tbh-1*) that transforms TA into OA (Alkema et al., 2005).

### 4.1 Tyramine metabolism and regulation

#### 4.1.1 Biosynthesis, transport, release, recycle, and breakdown

In flies and worms, TA biosynthesis is initiated by the decarboxylation of tyrosine by tyrosine decarboxylase (TDC) (Roeder, 2005; Huang et al., 2016) and encoded by the gene *Tdc* in *Drosophila* and by *tdc-1* in worms (Figure 4; Table 1). Importantly, TA serves as a precursor for the production of OA (Figure 4; Table 1). Interestingly, TA synthesis is regulated by the feeding state of the fly and acts as a signal for when the animal is sated (Cheriyamkunnel et al., 2021). Once synthesized, the neurotransmitter is packaged into synaptic vesicles by VMAT. *Drosophila* DAT, although sensitive to cocaine, appears to be less efficient in transporting OA and 5-HT (Porzgen et al., 2001; Wu and Gu, 2003). In mammals, TA is metabolized by MAO. In contrast to the mechanisms of inactivation found in vertebrates, MAO appears to be of limited importance in *Drosophila* (Roeder, 2005).

Similar to flies, TA synthesis in *C. elegans* begins with the conversion of the amino acid tyrosine to TA by TDC (encoded by the gene *tdc-1* in worms) (Figure 4; Table 1) and is terminated by the conversion of TA to OA by TA beta-hydroxylase (encoded by *tbh-1*) (Alkema et al., 2005). The catabolism of TA and OA are not fully understood, but it appears that N-acetylation and N-succinylation are competing mechanisms of biogenic amine metabolism (Artyukhin et al., 2013). TA is a substrate for VMAT, which has 47% similarity to human VMAT1 and 49% similarity to human VMAT2 (Duerr et al., 1999). As previously discussed, MAO has also been detected in *C. elegans*, suggesting that it could mediate the breakdown of TA and OA (Pirri et al., 2009).

### 4.2 Tyramine receptors

One prominent feature of the tyraminergic system is its receptor classes, which include TAR1, the first type of TA receptors present in *Drosophila*, previously known as “OctTyrR” class. TAR1 shows preference for TA binding over OA (Bayliss et al., 2013) and inhibits adenylyl cyclase that results in lower cAMP production levels. It also leads to the elevation of intracellular Ca^2+^ levels (Robb et al., 1994; Zhang and Blumenthal, 2017). The TAR2 family of receptors is known to be highly selective for TA and coupled to a release of calcium (Bayliss et al., 2013). A third TA receptor present in *Drosophila*, TAR3, when stably expressed in CHO cells, exhibited diminished levels of cyclic AMP, indicating its role in the inhibition of adenylyl cyclase. Similar to TAR2, it has been found to be associated with Ca^2+^ release. Both TAR2 and TAR3 share a 61% sequence identity and 79% sequence similarity (Bayliss et al., 2013), but they differ in tissue expression patterns in *Drosophila* (Flybase.org). TAR2 is abundant in the brain, thoracoabdominal ganglion and in the midgut of the adult fruit fly, whereas the orthologous TAR3 is highly expressed in the adult eye and in the tubule and hindgut of larvae. The Malpighian tubules, which are the primary urine production organs, are the best characterized system for studying tyraminergic signaling in native fly tissue.

In *C. elegans*, SER-2 (encoded by *ser-2*), TYRA-2 (encoded by *tyra-2*), TYRA-3 (encoded by *tyra-3*), and LGC-55 (encoded by *lgc-55*) are the tyraminergic receptors that have been discovered thus far. SER-2, TYRA-2, and TYRA-3 are GPCRs and TA appears to interact with the Gα subunit of each (Rex et al., 2005; Wragg et al., 2007; Donnelly et al., 2013). LGC-55 is a ligand-gated chloride channel (Ringstad et al., 2009), which is found in neurons and muscles receiving synaptic inputs from RIM (Pirri et al., 2009). In aversive conditions TA suppresses neck muscles needed for head movement and modulates AVB sensory neurons, resulting in reversed motion in which there is little head movement (Pirri et al., 2009).

SER-2 is found in GABAergic motor neurons and head muscles. It is coupled to the GOA-1/Gα_i/o_ pathway inhibiting GABA release on ventral body wall muscles (Donnelly et al., 2013) and bears similarities to mammalian α(2)-adrenergic receptors inhibiting neurotransmission release during fight or flight. These receptors are activated extra-synaptically with no innervation from RIM (Donnelly et al., 2013). SER-2 plays an important role in early development where it is needed for aversion memories in the L1 stage. In this pathway, AIB interneurons detect TA from RIM needed for memory formation (Jin et al., 2016). These receptors are also found in the RME motor neurons in the head and they are activated during escape behavior (Kagawa-Nagamura et al., 2018).

TYRA-2, located on neurons in the pharyngeal region, was discovered as a receptor with highest affinity for TA over other amines (Rex et al., 2005). TYRA-2 Gα_i/o_ receptor (upon binding to TA) was later found to regulate feeding rates through a blockage of 5-HT release. The behavior is exhibited when the worms come across inedible food (Fu et al., 2018). Furthermore, when TYRA-2, located on the ASH nociceptive neuron, detects a pheromone related to hunger, it elicits an aversive response in the worm (Chute et al., 2019). TYRA-3, which has multiple isoforms (Bendesky et al., 2011), is expressed in CEP and ADE neurons, allowing TA to modulate responses to aversive smells with reduced head movement through a pathway that is specific for TA and not OA (Wragg et al., 2007). TYRA-3Rs are found throughout the worm, including on the nociceptive ASH sensory neuron. This localization allows for a TA role in modulating pain (Hapiak et al., 2013).

### 4.3 Tyramine and behavior

#### 4.3.1 Drosophila

TA modulates a broad spectrum of behaviors across different insect species. These include effects on egg melanization (mosquito), sex pheromone production (moth), appetite (blowfly), and muscular contractions (locust) (Zhang and Blumenthal, 2017).

##### 4.3.1.1 Locomotion

TA and OA have opposite effects on the modulation of locomotion in *Drosophila* larvae (Saraswati et al., 2004). TA decreases crawling, whereas OA augments the insect’s locomotor activity. Interestingly, TA also contributes to regulation of normal and cocaine-induced locomotion. Flies lacking TA and OA due to a mutation in *Tdc2* exhibit hypersensitivity to an initial dose of cocaine and reduced basal locomotor activity (Hardie and Zhang, 2007). Conversely, the flies with a null mutation in *tßh* exhibit normal locomotor activity and cocaine response (Hardie and Zhang, 2007).

##### 4.3.1.2 Flight

For a long while, flight behavior was believed to be modulated strictly by OA. More recent research has revealed the synergism between OA and TA (Brembs et al., 2007). One interesting study revealed that flies lacking OA, and with their tyraminergic receptors blocked, showed wild-type-like flight behavior. The blockage of TARs did not increase the flight initiation and maintenance, indicating that TA inhibits flight behavior only at abnormally high TA levels. It also indicated that OA is not required for flight initiation (Brembs et al., 2007).

##### 4.3.1.3 Courtship

TA is involved in the regulation of other behaviors as well. When the tyramine-specific receptor is knocked out, it leads to a significant elevation in courtship activity between males in the absence of females, indicating an *in vivo* role for TyrR as an inhibitory component in courtship activity modulation. The data also show that TyR-expressing neurons control overall male sexual drive, highlighting a function of TA as an important neuromodulator of male courtship drive (Huang et al., 2016). TA is also involved in starvation response (Schützler et al., 2019), although one important caveat is the need to tease out an OA vs. a TA effect.

#### 4.3.2 C. elegans

##### 4.3.2.1 Touch response and escape behavior

One well-characterized role for TA is in the coordination of touch response. TA, through the action of the tyraminergic RIM motor neurons, has long been known (including from seminal work done in Sydney Brenner’s lab) to play a key role in linking the neural circuits that regulate locomotion and head movement (White et al., 1986). Upon anterior touch, *C. elegans* exhibit a backward movement (reversal) in which head movement is inhibited and regulated by TA (Alkema et al., 2005), through the activation of TA-gated chloride channel LGC-55; allowing the neurotransmitter to act as an inhibitory neurotransmitter (Pirri et al., 2009) (see TA receptors above). Interestingly, the neural circuitry of reversal movement is such that TA released from RIM neurons inhibits GABAergic RME motor neurons through a downregulation of calcium in RMEs (Kagawa-Nagamura et al., 2018). This circuitry is salient in the context of avoidance of predation—TA synthesis mutant *tdc-1* exhbits defects in escape from the constricting rings of the parasitic fungi *Drechslerella doedycoides* that trap worms (Maguire et al., 2011).

##### 4.3.2.2 Egg-laying

Both OA and TA have a role in egg-laying and because both mutants lack OA, it can be a challenge to tease apart the function of each neurotransmitter. However, investigators using genetic analyses demonstrated that while TA mutants were hyperactive in egg-laying, OA mutants made fewer eggs (Alkema et al., 2005), and in further studies showed that TA had an inhibitory role in egg-laying independent of OA.

## 5 Octopamine

OA, widely considered the fly homolog of norepinephrine, plays multiple roles during development and in the regulation of a variety of behaviors from larvae through adulthood (Monastirioti, 1999; Saraswati et al., 2004; Fox et al., 2006; Lawal et al., 2014). In behavioral circuits like locomotion in which multiple monoaminergic neurotransmitters are involved, OA plays a dominant role (Pizzo et al., 2013). In other cases, OA acts in an antagonistic fashion, such as with TA to regulate aspects of locomotion (Saraswati et al., 2004). Importantly, OA also plays a key role in the regulation of the central pattern generator in *Drosophila* and other insects (Fox et al., 2006; Buhl et al., 2008; Vierk et al., 2009). In *C. elegans*, OA is found in RIC interneurons which appear to form both chemical synapses and gap junctions (Alkema et al., 2005). RICs are part of a neural circuit that includes serotonergic ADF chemosensory neurons and dopaminergic CEP neurons that transmits odor signals (Zhang et al., 2021). In another serotonergic circuit, 5-HT binds to RIC neurons initiating the release of OA to avert the worm from food (Harris et al., 2011).

### 5.1 OA metabolism and regulation

#### 5.1.1 Biosynthesis, transport and release

OA is synthesized from TA by TβH (for review, see (Monastirioti, 1999)) (Figure 4; Table 1; see section above). Like the other monoamines, once synthesized, OA is packaged into synaptic vesicles for subsequent exocytosis by VMAT. Like TA, OA serves as an antagonist to 5-HT. Initial experiments showed that when applying exogenous 5-HT and OA separately, opposite behavioral responses were seen for pharyngeal contraction (a key means through which worms ingest bacteria) and egg-laying, with an increase of both behaviors for 5-HT and a decrease for OA (Horvitz et al., 1982). Both neurotransmitters also regulate other aspects of pharyngeal action. One group has reported that 5-HT decreases the duration of a pharyngeal action potential (and can only do so with functioning MC and M3 motor neurons) while OA has the opposite effect (Niacaris and Avery, 2003), allowing the worm to adjust to food availability or lack thereof.

### 5.2 Octopamine receptors

The discovery of the first *Drosophila* OA receptor, OAMB (Han et al., 1998) was consequential in uncovering the mechanisms underlying the regulation of the octopaminergic signaling. OAMB, also known as Octα1R, along with the later discovered Octα2R, belongs to the family of *α*-adrenergic-like GPCRs. Based on sequence identity, a group of three other OA GPCRs with structural similarities to ß-adrenergic receptors have been identified: Oct1ßR, Oct2ßR, and Oct3ßR (El-Kholy et al., 2015). Both *α*- and ß-adrenergic OA receptors show preference for OA binding, but may also be activated by TA (Bayliss et al., 2013). Expression analysis revealed that they are abundant in the CNS. Moreover, strong Octß1R expression is seen in adult CNS, specifically in the pars intercerebralis, MBs, and optic lobes. Similar to OAMB and Octß1R receptors, the MB is the most obvious organ in which Octß2R is expressed. Octß3R is similarly well expressed in MBs (El-Kholy et al., 2015).

Three OA receptors have been identified in *C. elegans*, SER-3 (encoded by *ser-3*), SER-6 (encoded by *ser-6*), and OCTR-1 (encoded by *octr-1*), each coupled to Gα_q_, Gα_q_ and Gα_i_, respectively (reviewed in (Mills et al., 2012a)). All 3 *C. elegans* OA receptors appear to be similar to mammalian *α*-adrenergic receptors (Mills et al., 2012b). SER-3 has been found to induce cAMP response element-binding protein (CREB) activation in SIA neurons, during starvation (Suo et al., 2006). When OA levels increase, the neurotransmitter binds to SER-3 on ASH, antagonizing OCTR-1. Moreover, when OA is increased, it also binds to ADL, AWB and ASI sensory neurons, which may cause a cascade of neuropeptide release activating receptors throughout the worm, possibly modulating locomotion based on the aversive response (Mills et al., 2012a).

#### 5.2.1 OA and behavior

##### 5.2.1.1 Drosophila

OA is a central regulator and modulator of numerous behaviors and physiological processes in invertebrates like *Drosophila.* These behaviors range from locomotion (see below) to the tracking of CO_2_ during flight (Wasserman et al., 2013). Lack of OA decreases the life span of adult flies, but substantially increases starvation resistance, indicating a key role for OA as a central mediator of stress response in invertebrates (Li et al., 2016).

###### 5.2.1.1.1 Locomotion

OA is the predominant neurotransmitter that regulates locomotion (Saraswati et al., 2004; Fox et al., 2006), playing a key role in baseline locomotion (Simon et al., 2009). Studies in *VMAT* null mutants, which have severely reduced locomotion ability, have shown that among the monoamines, OA alone is necessary and sufficient to restore locomotion activity (Chen et al., 2013). In circuits that control stimulated locomotion, OA is particularly important in the regulation of the central pattern generation (CPG), a semi-autonomous circuit that directs repetitive motion (Fox et al., 2006; Brembs et al., 2007). Important findings demonstrate that OA dominates over DA in regulating locomotion, with DA playing a more modulatory role vis-à-vis OA (Chen et al., 2013; Pizzo et al., 2013). Despite the extensive involvement of OA in locomotion regulation, its precise circuitry in *Drosophila* remains poorly understood. In the larval NMJ, while the role of OA is well-defined and some boutons are known to be aminergic, it remains unclear how the locomotion circuit is wired at points in between the CNS and the NMJ.

###### 5.2.1.1.2 Learning and memory

OA is important for both appetitive and aversive learning, mirroring the effect of DA and indeed highlighting an indispensable interaction between both systems (Sabandal et al., 2020). Interestingly, OA action through a localization-specific expression of Octβ1R differentially drives each type of olfactory learning in *Drosophila.* Octβ1R expression in the αβ neurons of the MB mediates aversive learning, while the expression of that receptor class in the projection neurons drives appetitive learning in a cAMP-dependent manner (Sabandal et al., 2020). *tβh* mutant flies that lack OA were also found to exhibit impairments in appetitive olfactory learning, further demonstrating the role of OA in learning behavior (Iliadi et al., 2017).

###### 5.2.1.1.3 Courtship and aggression

In *Drosophila* males, OA depletion leads to a suppression of courtship conditioning (Zhou et al., 2012). Moreover, OA modulates the choice between courtship and aggression. A competitive male courtship assay showed that males with no OA (null mutant) or with low OA (hypomorph) do not adapt to alterations in sensory cues and court both sexes (Certel et al., 2007). Studies using synaptic blockade and excitation approaches in subsets of the doublesex-positive neuronal cluster, pC1 in *Drosophila* have demonstrated that the *fruitless (fru)*-negative subfraction of that cluster (a group of about twenty cells) serves as an aggression-triggering complex, whereas *fru* positive subfraction acts as the courtship-triggering center (Koganezawa et al., 2016). Moreover, research done on females lacking OA showed a disruption in postmating response in mated females and an increase in OA induced postmating response in virgin females (Rezával et al., 2014). Furthermore, OA modulates *Drosophila* aggression (Hoyer et al., 2008), as amply demonstrated in Baier et al. (2002) The Brembs lab, which used a *t*ß*h* mutant along with wild type animals, observed a decrease in aggressive behavior in the mutant. Additionally, mutant flies which lacked OA showed an increase (up to ten fold) in TA levels (Monastirioti, 1999).

###### 5.2.1.1.4 Egg-laying

OA has long been known to be involved in egg-laying and other aspects of the female reproductive circuit. Egg-laying deficits of TβH flies are partially rescued by feeding them OA (Monastirioti, 1999). OA signaling is also required for egg retention and release (Chen et al., 2013). OA increases the strength of contractions of the peritoneal sheath, which stimulates the ovulation of the egg. At the same time, it relaxes the oviduct enabling egg release. Therefore, egg-laying deficits in flies lacking OA may be explained using the model that OA is likely to be released during egg-laying behavior to augment the strength of ovarian contractions and to relax the oviduct to accelerate the ovulation (Middleton et al., 2006).

###### 5.2.1.1.5 Response to starvation stress

Given that starved animals often exhibit elevated locomotion, a study found that *tβh* null mutants showed no increase in locomotor activity upon starvation, suggesting that OA regulates starvation-induced enhanced activity in adult flies (Yang et al., 2015). Moreover, there has been great interest in understanding how starvation influences the fly’s decision making. In the context of starvation response, it is important to note that both OA and TA play a role in starvation-induced sugar responsiveness. *tßh* mutants, which are unable to synthesize OA showed reduced sugar response upon starvation, indicating the requirement of OA for food seeking (Damrau et al., 2017). One important caveat is that *tßh* mutants also have elevated TA levels, raising the need to tease out an OA vs TA effect.

###### 5.2.1.1.6 Sleep

OA has also been implicated in the regulation of sleep, acting through neuronal protein kinase A to decrease sleep activity. Flies with mutations in OA synthesis genes show a phenotype of increased sleep (Crocker and Sehgal, 2008). Additionally, the identification of circuits marking the wake-promoting effects of OA in *Drosophila* has led to further recognition of ASM cells in the medial photocerebrum as the wake-promoting octopaminergic cells. It has also been stated that effects of OA on sleep:wake mechanisms are mediated by the cAMP dependent isoform of OAMB receptor (Crocker et al., 2010). This effect mirrors that of norepinephrine, its mammalian analog, which is also known to promote awake states (Mitchell and Weinshenker, 2010).

###### 5.2.1.1.7 Vision

OA plays an interesting and multivariate role at all tested levels of visual processing, including in the modulation of motion vision (for review, see (Cheng and Frye, 2020)). Moreover, octopaminergic neurons are both necessary and sufficient for the boost seen in the response of flies to visual motion (Suver et al., 2012), a role that distinguishes that neurotransmitter from 5-HT, which has a more limited function in modulating peripheral vision. Further, OA is needed for fast-moving behavioral responses to fast-moving visual stimuli but not for slow-moving ones (Strother et al., 2018). The Frye lab has uncovered one intriguing function of OA, which appears to be to reverse the normal visual avoidance of an object by the fly in response to an odor, triggering OA release based on the activity of its receptors (Cheng et al., 2019).

##### 5.2.1.2 C. elegans

The interaction between OA and DA is particularly important during aversive conditions like starvation and noxious odors. Monoamines can activate the transcription factor, CREB, initiating transcription at promoters with the cAMP response element. Under conditions of food scarcity, OA is released, activating CREB through EGL-30, a Gα_q_, which probably works through SER-3 in SIA neurons (Suo et al., 2006). DA conversely is released when food is present; it binds to DOP-3 on the RIC neurons which couples with Gα_i/o_, suppressing not just OA release from RIC neurons, but ultimately preventing CREB activation in SIA neurons (Suo et al., 2009). When worms avoid certain repulsive odors after pre-exposure, DA will bind to DOP-3 in the RIC interneurons, likely following the pathway for starvation described above (Kimura et al., 2010).

## 6 Histamine

Histamine is the widely used neurotransmitter in insect photoreceptors and is known for its role in promoting wakefulness and circadian rhythm regulation. Histamine also accumulates in putative neurosecretory cells and in the interneurons innervating an extensive portion of the body ganglia (Monastirioti, 1999). In contrast to other flies, *Drosophila* accumulates histamine in the PNS, including in the mechanosensory neurons of hair sensilla on the imaginal cuticle (Monastirioti, 1999). The tissue content of histamine and carcinine is highest in the eyes, followed by the brain and cuticle (Denno et al., 2016).

### 6.1 Histamine metabolism and regulation

**FIGURE 5:**
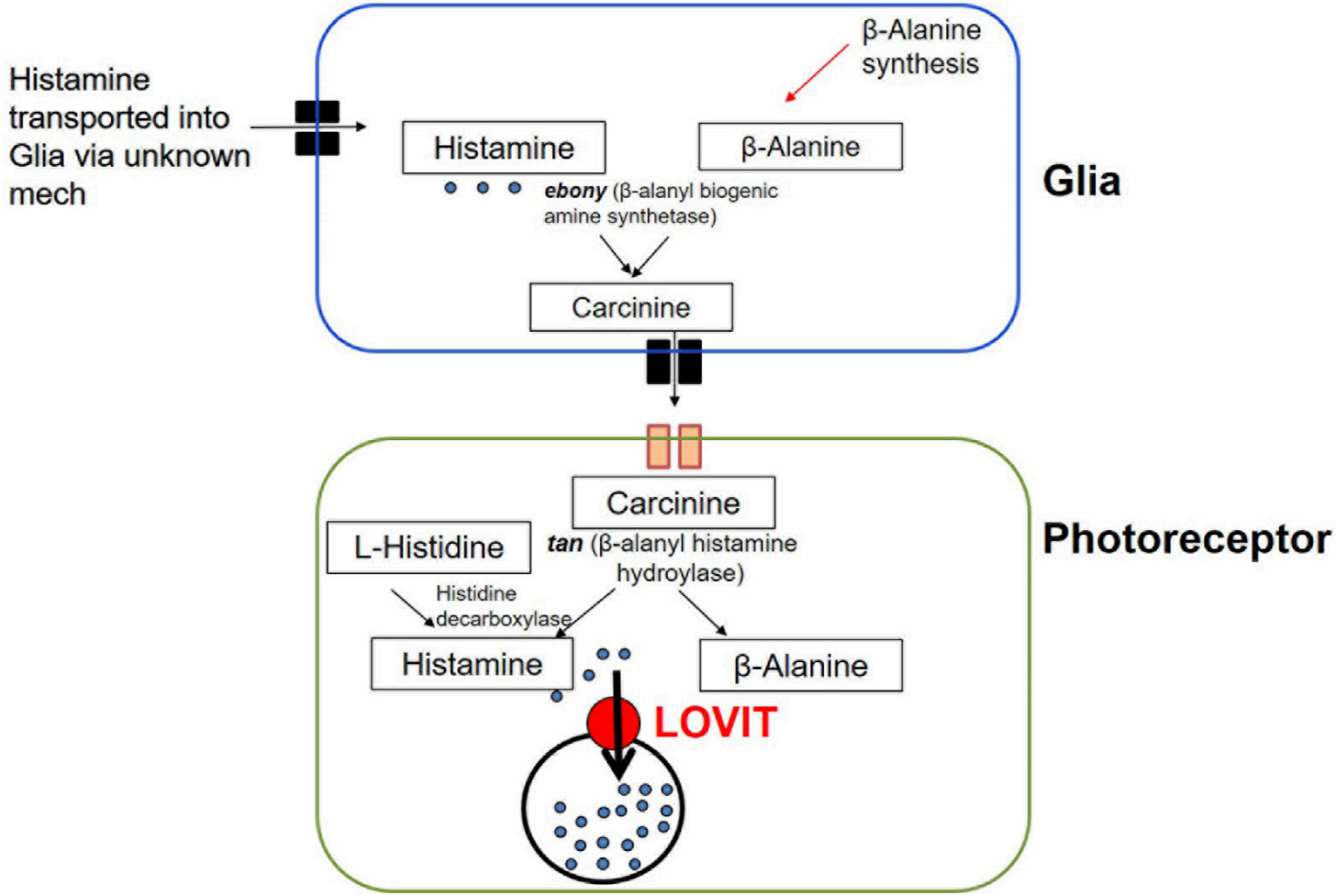
Histamine synthesis and recycling in Drosophila. Histamine is synthesized *de novo* from L-histidine in photoreceptors by histidine decarboxylase. Histamine is also cycled through glia first by conversion into carcinine by *ebony* after which it is transported out of glia (through an unknown receptors) and into photoreceptors through CarT. Carcinine is then converted back into histamine by *tan*. Histamine is subsequently transported into synaptic vesicles by LOVIT.

*Biosynthesis, transport, release, recycle and breakdown:* Histamine is the primary neurotransmitter released in insect photoreceptors (Hardie, 1989). Histidine decarboxylase (encoded by *hdc*) is responsible for the *de novo* biosynthesis of histamine from L-histidine at presynaptic release sites (Figure 5; Table 1) (Melzig et al., 1998). The precursor L-histidine is transported into histamine-synthesizing cells by the histidine transporter, TADR (torn and diminished rhabdomeres) (Han et al., 2022). Once produced, histamine is packaged and transported into endocytic vesicles. Two transporter proteins help facilitate this role. VMAT-B is a splice variant of the transporter and is expressed in a small subset of laminal glia in the fly optic lobe (Romero-Calderon et al., 2008). In photoreceptors, LOVIT is a putative synaptic vesicular transporter that is essential for the packaging and transport of histamine ((Xu and Wang, 2019); for review, see (Deshpande et al., 2020)). It is localized in photoreceptor synaptic vesicles and a defect in its function disrupts visual neurotransmission (Xu and Wang, 2019). Histamine recycling from glial cells into photoreceptors is mediated by Carcinine Transporter (CarT) (Xu et al., 2015) (Figure 5; Table 1). Briefly, histamine is transported into glial cells through an unknown mechanism and converted into the carcinine, a product also known as *ß*-alanyl histamine. This reaction is catalyzed by N- *ß*-alanyl dopamine synthase, encoded by *ebony* in *Drosophila* (*ebony* is also known to convert DA and other biogenic amines) (Denno et al., 2016). Carcinine is then transported out of the glia through an unknown transporter, after which it is taken up by CarT into photoreceptors for reconversion into histamine by *ß*-alanyl histamine hydroylase, encoded by *tan* (Deshpande et al., 2020)*,* a gene that was first identified in *Drosophila* 17 years ago (True et al., 2005). Because the rate of histamine synthesis is low, the conversion of carcinine back to histamine in the photoreceptors is an important step in regulating the levels of histamine available for neurotransmission.

### 6.2 Histamine receptors

Two genes encoding histamine-gated chloride channels, *ora triansentless* (*ort*) and histamine-gated chloride channel subunit 1 (*HisCl1*) have been identified in *Drosophila*. The latter receptor has been shown to act in photoreceptor neurons to synchronize flies’ behavioral rhythms with light-dark cycles (Alejevski et al., 2019). It also regulates wake-promoting signals. Moreover, the defects in the genes encoding the proteins involved in histamine signaling (*HisCl1, ort*) cause abnormal temperature preference behaviors (Hong et al., 2006).

### 6.3 Histamine and behavior

It follows from the key role that histamine plays in the *Drosophila* visual system that the HisCl1 histamine receptor acts in Rh6-expressing retinal photoreceptors to synchronize the fly’s behavioral rhythms with light-dark cycles (Alejevski et al., 2019). Importantly, knockdown of LOVIT in photoreceptors disrupts visual synaptic transmission, as measured by ON and OFF transients of an electroretinogram at the start and end of an electrical stimulation (Xu and Wang, 2019). Moreover, an RNA interference knockdown of LOVIT impairs phototaxis, while a photoreceptor neurons specific expression (using GMR > Gal4) restored both defects in the ON and OFF transients and phototaxis seen in LOVIT-RNAi knockdown animals (Xu and Wang, 2019).

## 7 Discussion

Studies on the biogenic amines in *Drosophila* and *C. elegans* bring to the fore some interesting topics that merit further discussion as presented below.

### 7.1 Case studies in antagonistic neurotransmitter interaction

Cooperative/antagonistic interaction between OA and TA manifest in several behaviors. Here, we describe a few. One case in point is the regulation of flight in *Drosophila*. Levels of OA and TA shape the action of the CPG (Brembs et al., 2007). OA is also required for the maintenance of flight but not for its initiation, while TA acts to inhibit flight maintenance at low OA levels. With respect to flight initiation, TA inhibits that step regardless of OA levels, highlighting a complex interaction between both neurotransmitters to regulate flight behavior (Brembs et al., 2007).

Further, countervailing actions of 5-HT and OA shape pharyngeal pumping action in *C. elegans* behavior*.* This raises the possibility that there are many more such examples of a complex interplay between monoamine neurotransmitters, as they modulate one another to define specific behavioral contexts. Therefore, the elucidation of those possible interactions represents another area of potential future interest.

The relative contributions of different biogenic amines to place memory formation are another interesting research area. Notwithstanding the well-established role for DA in associative learning, initial evidence pointed to 5-HT, not DA, as necessary for place memory in the fruit fly (Sitaraman et al., 2008). However, more recent evidence from the same group complicates that finding somewhat, by demonstrating the involvement of a cluster of DA neurons in heat-box place learning (Mishra et al., 2020). This update to the body of knowledge opens doors for follow up studies aimed at identifying what, if any, interactions exist between both neurotransmitter systems during place memory modulation.

### 7.2 DAT/D2R interaction. Is there more than meets the eye?

The functional consequences of a D2 receptor-DAT interaction have been shown in *C. elegans* (Dopamine section above) and are consistent with work in rodents showing that DAT forms a protein complex with D2R and that its cell surface expression levels are regulated by that interaction (Lee et al., 2007). The nature of this interaction represents an interesting area of ongoing research in the field. Given the importance of both proteins for the maintenance of DA homeostasis, their likely physical interaction raises many interesting questions, including a full delineation of the biological relevance of the protein complex, and the catalog of other interactors that may stabilize or disrupt that complex. Along those lines, a peptide that disrupts that D2R-DAT complex has been identified and found to protect against DA neurotoxicity (Su and Liu, 2017). The abiding strengths of *Drosophila* and *C. elegans* as genetic models make them powerful tools of performing genetic analysis that may help uncover more such interactors.

### 7.3 DAT and VMAT, do the twain meet to regulate DA synaptic release?

There has been much discussion regarding the central, oppositional roles that DAT and VMAT play in the regulation of cytosolic DA levels and ultimately extracellular DA. Much of this is the subject of a fine review (see (Mulvihill, 2019)). It should be noted that findings in flies and worms strongly corroborate these transporters’ roles in maintaining DA homeostasis (see Section 2). However, one intriguing unanswered question is whether a type of direct interaction exists to bridge these two forms of synaptic DA regulation. It is certainly plausible, given the D2R mediation of DAT endocytosis, and the trafficking of VMAT back into the cytosol following SV fusion, that both transporters could be expressed on the same endocytic vesicles.

### 7.4 The future of LOVIT

An exciting potential new ground lies on the follow up to the discovery of LOVIT as a VMAT-independent mechanism for the transport of histamine. Its localization to photoreceptors indicates a narrow cell-type expression, unlike VMAT, which is found in multiple aminergic neuron-types. However, it will be interesting to know if there are other organisms or monoaminergic systems that use this transporter in a similar fashion.

## 8 Summary

Biogenic amines play a myriad of roles in the regulation and modulation of neuronal functions in *Drosophila*, *C. elegans* and other invertebrates. In many of these roles, the neurotransmitters not only affect behaviors, but they also regulate the actions of other biogenic amines, sometimes in a cooperative fashion and at other times in an antagonist sense. A plethora of new knowledge has been uncovered from work in both model systems, much of it paving the way for interesting insights into the diverse roles that monoamine neurotransmitters play in the regulation of essential life processes, and in how the molecules themselves are regulated.
